# Optimizing water and nitrogen productivity of wheat and triticale across diverse production environments to improve the sustainability of baked products

**DOI:** 10.3389/fpls.2022.952303

**Published:** 2022-09-02

**Authors:** Santiago Tamagno, Cameron M. Pittelkow, George Fohner, Taylor S. Nelsen, Joshua M. Hegarty, Claudia E. Carter, Teng Vang, Mark E. Lundy

**Affiliations:** ^1^Department of Plant Sciences, University of California, Davis, Davis, CA, United States; ^2^California Grain Foundation, Woodland, CA, United States; ^3^California Wheat Commission, Woodland, CA, United States; ^4^Division of Agriculture and Natural Resources, University of California, Davis, Davis, CA, United States

**Keywords:** sustainability, nitrogen, water, agri-food chain, baking industry, nitrogen use efficiency

## Abstract

Wheat (*Triticum aestivum* L.) is a major global commodity and the primary source for baked products in agri-food supply chains. Consumers are increasingly demanding more nutritious food products with less environmental degradation, particularly related to water and fertilizer nitrogen (N) inputs. While triticale (× *Triticosecale*) is often referenced as having superior abiotic stress tolerance compared to wheat, few studies have compared crop productivity and resource use efficiencies under a range of N-and water-limited conditions. Because previous work has shown that blending wheat with triticale in a 40:60 ratio can yield acceptable and more nutritious baked products, we tested the hypothesis that increasing the use of triticale grain in the baking supply chain would reduce the environmental footprint for water and N fertilizer use. Using a dataset comprised of 37 site-years encompassing normal and stress-induced environments in California, we assessed yield, yield stability, and the efficiency of water and fertilizer N use for 67 and 17 commercial varieties of wheat and triticale, respectively. By identifying environments that favor one crop type over the other, we then quantified the sustainability implications of producing a mixed triticale-wheat flour at the regional scale. Results indicate that triticale outyielded wheat by 11% (*p* < 0.05) and 19% (*p* < 0.05) under average and N-limited conditions, respectively. However, wheat was 3% (*p* < 0.05) more productive in water-limited environments. Overall, triticale had greater yield stability and produced more grain per unit of water and N fertilizer inputs, especially in high-yielding environments. We estimate these differences could translate to regional N fertilizer savings (up to 555 Mg N or 166 CO_2_-eq kg ha^−1^) in a 40:60 blending scenario when wheat is sourced from water-limited and low-yielding fields and triticale from N-limited and high-yielding areas. Results suggest that optimizing the agronomic and environmental benefits of triticale would increase the overall resource use efficiency and sustainability of the agri-food system, although such a transition would require fundamental changes to the current system spanning producers, processors, and consumers.

## Introduction

During the last decades, intensification of agricultural systems in response to global food demand has increased the environmental footprint of food production with direct consequences on freshwater and landscape degradation (Vermeulen et al., 2012). Agriculture consumes nearly 70% of global freshwater resources (FAO, 2010), and it is the primary source of freshwater degradation due to nitrate leaching losses (Fowler et al., 2013). Likewise, agri-food supply chains are a major contributor to nitrogen (N) losses to the environment. For instance, it is estimated that 40% of the total environmental impact of producing a loaf of bread derives from inefficiencies in N fertilizer use in the cultivation phase (Goucher et al., 2017). More recent work evaluating bread supply chains in China found that wheat cultivation produced 77% of total greenhouse gas (GHG) emissions but less than 8% of the total economic benefits (Deng et al., 2021). With the projected increase of human population and changes in dietary patterns in the coming years, the challenge to produce food using less water and N has never been more urgent (Springmann et al., 2018). Growing environmental concerns over agricultural pollution have raised the interest in marketing food products on the basis of sustainability metrics, creating opportunities to promote better agricultural resource use efficiency.

Wheat (*Triticum aestivum* L.) plays a major role in global agri-food chains as it represents the largest cultivated crop (FAOSTAT, 2021) and a main source of calories for the human population (Shewry and Hey, 2015). In California (United States; US), common wheat is grown under a range of semiarid environments including rainfed, partially-and fully-irrigated production systems, and terminal drought impacts on crop productivity are frequently observed. The main limitation to productivity is therefore generally related to water availability, but there are important interactions with soil N supply (Cossani et al., 2010; Sadras et al., 2016), with a range of possible mechanisms than can lead to crop water or N stress, depending on the circumstances. Under rainfed conditions, highly variable soil water dynamics lead to unpredictable and often yield-limiting late-season water and N availability. In contrast, when crop yield potential is high due to sufficient water (supplied *via* irrigation or ample precipitation), soil N supply often becomes most limiting to yield, providing rationale for applications of N fertilizer in excess of crop N removal, which has negative consequences for sustainability.

Intra-and inter-annual rainfall is highly variable in the region (Dettinger et al., 2011; Pathak et al., 2018), and this increases the occurrence both of excessive water availability and periodic drought, each of which can reduce overall water use efficiency. Excess water reduces N use efficiency of pre-plant N fertilizers when water volumes exceed crop N uptake, moving N fertilizer below the rootzone. Likewise, when water and N availability are non-limiting early in the season, vigorous crop growth can deplete soil water reserves with negative impacts in later, more water-demanding developmental stages (i.e., anthesis and grain filling; van Herwaarden et al., 1998; Calviño and Sadras, 2002) when rainfall frequency is lower. Because water and N are co-limiting resources (Cossani and Sadras, 2018), under drought conditions, water limitations also limit plant N uptake, compounding drought stress with crop N stress.

Within this context, wheat production environments in California span from low-yielding conditions, usually limited by water availability, to high-yielding conditions, typically in irrigated fields where yield potentials are often limited by mineral N availability. Both extremes require the efficient management of N and water to achieve profitable grain yields while limiting input use and associated negative externalities. Many farmers alternate wheat with triticale (*× Triticosecale*) due to its generally superior yields and the regional demand for animal feed both as forage and grain. Within agronomic literature, triticale is often referenced as having superior abiotic stress tolerance compared to wheat (Blum, 2014), and it has shown better performance in paired experiments with same amounts of water and N inputs suggesting a higher water productivity and N use efficiency (Giunta et al., 1993; Estrada-Campuzano et al., 2012; Roques et al., 2017). However, other studies have reported yield advantages in wheat over triticale (Sinha et al., 1986; Ellen, 1993; Gutteridge et al., 1993). This inconsistency across literature could be associated with the complex water-N relationships described above, the lack of genotypic variability represented in these trials, or the number of environments tested. Further research is required to compare yield stability and resource use efficiency of triticale to wheat under variable conditions of water and N stress. Understanding which crop type does best in different environments has the potential to improve productivity at the field-level while also presenting options for targeted production to optimize crop productivity per unit water and N fertilizer input at the regional level.

Although triticale has attracted attention for its agronomic characteristics, its potential as a milling product is more limited. Unlike wheat, triticale breadmaking properties are weaker due to inferior gluten content and quality (Mergoum et al., 2004). Indeed, use of triticale flour for leaven bread has been hindered by the weak elasticity and in some cases stickiness of its dough, which together complicate its use for high-speed mixing used by large volume industrial bakeries. Therefore, attempts to use triticale flour in baking applications have generally resulted in poor quality dough unless blended with wheat flour. As such, different blending ratios containing up to 60% of triticale flour have been used to improve bread quality (Bakhshi et al., 1989; Naeem et al., 2002). In spite of its baking attributes, triticale has some attractive traits from a nutritional standpoint, including properties such as dietary fiber or amino acid composition (Mosse et al., 1988; Onwulata et al., 2000). To justify market development efforts in response to increasing consumer interest in nutritional value and sustainability of food products, triticale production must provide desirable consumer attributes such as pleasing flavor and potential health benefits that are distinct from wheat. It must also achieve high resource use efficiency and productivity compared to wheat.

Given previous work indicating the superiority of triticale productivity compared to wheat growing under similar conditions, we hypothesize that increasing the proportion of triticale in the baking supply chain could reduce the environmental footprint for water and N fertilizer use in California. Despite the common perception of triticale as more stress-tolerant than wheat, there are few studies comparing agronomic differences between wheat and triticale for a wide range of commercial genotypes after isolating the effects of water and N stress in parallel field experiments covering different environments. Thus, the overall objective of this study was to quantify the relative grain productivity and protein concentration of triticale and common wheat crop types across diverse Mediterranean environments in California. More specifically, we identified environmental conditions that favor one crop type compared to the other and quantified sustainability implications of a mixed triticale-wheat flour in terms of water and N productivity at the regional scale.

## Materials and methods

### Field experiments

The dataset was comprised of statewide field trials comparing commercial varieties and advanced breeding lines of common wheat and triticale cultivars between 2017 and 2020 by the University of California Small Grains Variety Testing Program. Field trials were conducted across a wide range of soil types (Supplementary Figure S1), environmental and management conditions, which included managed N and water stress trials (Table 1). Water-stress trials were either rainfed or received a minimal amount of early-season irrigation to create terminal drought conditions and were fully fertilized with N. In contrast, N-stress trials were not fertilized with mineral N but were irrigated to avoid drought stress. From the 37 site-years comprising the dataset, some trials received supplementary irrigation when necessary whereas others did not. The 27 site-years in the “average” condition (Table 1) were grown on commercial farms according to grower practices or on research stations where regional best management practices were used. These sites represent the range of conditions and management practices in the state which can sometimes include limited water inputs. A randomized complete block design with four replications was used at all trial locations. Plots were sown to target 300 seeds m^−2^ which resulted in average seeding rates of approximately 120 kg ha^−1^. Each plot was six or nine drill rows wide (0.12 to 0.23 m row spacing) and 3.9 to 5.5 m long.

**Table 1:** Total water supply, N fertilizer range, number of environments and total number of wheat and triticale genotypes used at each treatment condition.

Treatments	Total water (mm)	N fertilizer (kg N ha^−1^)	Environments	Genotypes	
				Wheat	Triticale
Average	247–1,381	(0)^a^ 9–280	27	67	17
N stress	400–839	–	6	67	17
Water stress	252–427	112–224	4	62	17

a One site with high residual soil N and organic carbon where no mineral N was applied.

### Crop measurements and sustainability metrics

At harvest, grain samples were harvested from all rows using an experimental plot combine. Grain yield (kg ha^−1^) was estimated, and subsamples from each plot were collected to measure grain moisture and protein concentration. Grain yields were standardized to 12% moisture content, and protein concentration was measured *via* NIR spectroscopy calibrated to total N measured *via* combustion. Protein yield is the product of grain yield by protein concentration (kg ha^−1^). Protein concentration was converted to total N using 5.81 conversion factor (Fujihara et al., 2008) and multiplied by grain yield (Eq. 1). Vegetative N uptake was calculated using 0.76 and 0.78 N harvest index for wheat and triticale, respectively (Eq. 2). Values correspond to an extensive literature report from (Geisseler, 2016) on N concentration in harvested crop parts with emphasis on California data. Total N uptake is the sum of grain N uptake and vegetative N uptake. Because removal of crop straw is the most common practice in the region for both crops, N use efficiency (NUE) was calculated as a ratio between N outputs (total N uptake) to N inputs (N fertilizer; Eq. 3). Crop water productivity and partial factor productivity of N (PFPN) were the amount of grain yield produced per unit of water (rainfall plus irrigation; Rodrigues and Pereira, 2009; Fernández et al., 2020) or N input (Ladha et al., 2005; Congreves et al., 2021) calculated as Eqs 4, 5, respectively.


Eq. 1 $$\mathrm{Grain}\;N\;\mathrm{uptake}\;\left(\mathrm{kg}\;N\;ha^{-1}\right)=\mathrm{Protein}\;\mathrm{yield}\times 5.81$$

Eq. 2 $$\begin{array}{cc} \begin{array}{l} N\;\mathrm{vegetative}\;\\ \mathrm{uptake}\;\left(\mathrm{kg}\;N\;ha^{-1}\right) \end{array} & =\frac{\mathrm{Grain}\;N\;\mathrm{uptake}}{N\;\mathrm{harvest}\;\mathrm{index}}-\mathrm{Grain}\;N\;\mathrm{uptake} \end{array}$$

Eq. 3 $$NUE=\frac{\mathrm{Total}\;N\;\mathrm{uptake}\;\left(\mathrm{kg}\;N\;ha^{-1}\right)}{N\;\mathrm{fertilizer}\;\left(\mathrm{kg}\;N\;ha^{-1}\right)}$$

Eq. 4 $$\begin{array}{cc} \begin{array}{l} \mathrm{Water}\;\mathrm{productivity}\;\\ \left(kg_{\mathrm{grain}}\;mm^{-1}\right) \end{array} & =\frac{\mathrm{Grain}\;\mathrm{yield}\;\left(\mathrm{kg}\;ha^{-1}\right)}{\mathrm{Water}\;\mathrm{supply}\;\left(\mathrm{mm}\;ha^{-1}\right)} \end{array}$$

Eq. 5 $$\mathrm{PFPN}\;\left(kg_{\mathrm{grain}}\;\mathrm{kg}\;N^{-1}\right)=\frac{\mathrm{Grain}\;\mathrm{yield}\;\left(\mathrm{kg}\;ha^{-1}\right)}{N\;\mathrm{fertilizer}\;\left(\mathrm{kg}\;N\;ha^{-1}\right)}$$

### Statistical analysis

Effects of N and water stress environments and their interactions were tested in an analysis of variance by fitting general linear mixed models to the entire dataset. Models were fitted in R software using the *lme* function from the *nlme* package (Bates et al., 2015). To quantify the differences between crop types across environments, we conducted an environmental index (EI) analysis (Finlay and Wilkinson, 1963), where the EI is the average yield of all varieties of wheat and triticale tested at each particular environment. Stability analysis has been previously used to compare triticale with other cereals (Josephides, 1992; Méndez-Espinoza et al., 2019) or to characterize crop types in other cultivated species (Salmeron et al., 2014; Tamagno et al., 2016).

**Figure 1:**
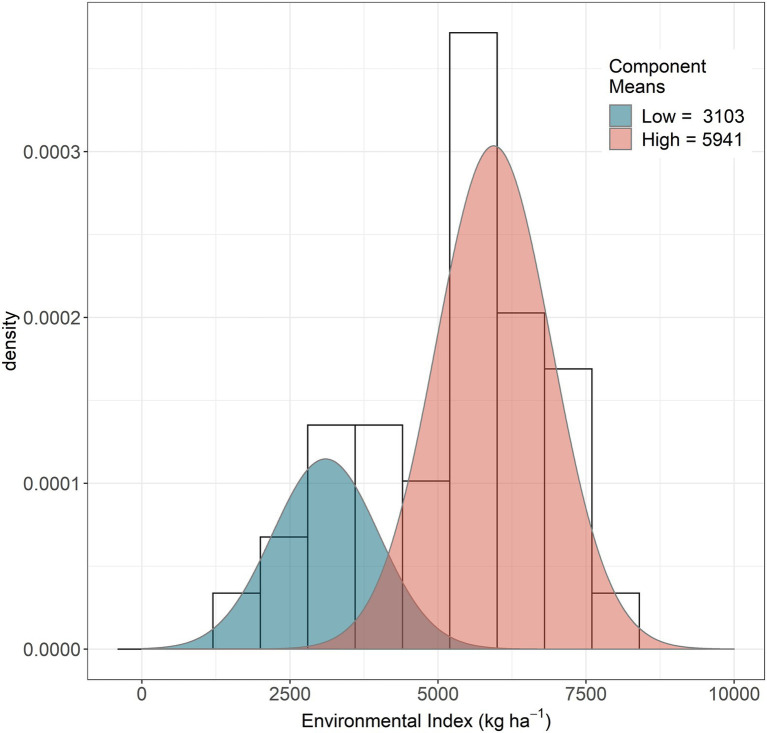
Frequency distribution and mean values (kg ha^−1^) for the low and high environmental index clusters.

We were interested in identifying crop type performance in low and high-yielding conditions. To determine each group of environments (low and high), we ran an expectation–maximization (EM) algorithm over the EI observations using the function *normalmixEM* from the R package *mixtools* (Benaglia et al., 2009). The iteration process produced low and high EI groups with mean values of 3,103 and 5,941 kg ha^−1^, respectively (Figure 1). The average between the groups was 4,522 kg ha^−1^. This value was used to define the limits of high and low-yielding environments. We determined which crop type performed best in each group in order to create different scenarios targeting those environments.

Based on this classification and the mean values for grain yield, protein concentration, water productivity, NUE and PFPN, different scenarios were analyzed to study the implications of targeting each crop type to all environments or to specific environments assuming a 40:60 blending ratio of flours (wheat:triticale). First, the average grain yield, protein concentration, and water and N metrics for each crop type were summarized for the entire dataset and for low and high-yielding environments (below and above 4,522 kg ha^−1^, respectively). Second, two scenarios were evaluated for each metric based on these averages: (1) A scenario using average values from the entire data set where weighted means were calculated on the basis of 60 and 40% for triticale and wheat, respectively for each variable. Thus, the final weighted mean would reflect the contribution of each crop for the flour blending target; (2) A second scenario with targeted production, using average values calculated as weighted means using 60% weight for triticale from the high-yielding environments and 40% for wheat from the low-yielding environments. This scenario assumes that triticale production is targeted to high-yielding environments and wheat to low-yielding environments to explore the potential for improving sustainability at the regional scale.

Wheat production across all environments was compared with the targeted production of wheat and triticale (second scenario), and differences in global warming potential (GWP) were calculated as kg of CO_2_ equivalents (CO_2_-eq) reduced through fertilizer savings. The N fertilizer required for each scenario was calculated based on the amount of wheat harvested for grain in California during the 2021 season (NASS-USDA, 2022) divided by the PFPN for each scenario. The difference between N requirements of each scenario was converted to CO_2_-eq using a factor of 10.15 kg CO_2_ eq kg N^−1^ which is the result of integrating 4.65 kg CO_2_ eq kg N^−1^ from direct N_2_O emissions (IPCC, 2006), 4 kg CO_2_ kg N^−1^ from fertilizer production and transport (Snyder et al., 2009) and 1.5 kg CO_2_-eq kg N^−1^ from indirect N_2_O emissions derived from NH_3_ volatilization and NO_3_ leaching losses. In this regard, methodology from IPCC (2006) suggest that 10% of N applied is lost as volatilization with 1% representing N_2_O-N emissions, whereas for NO_3_ leaching these values are 30 and 0.75%, respectively. Then, N_2_O emissions were calculated as N_2_O = N_2_O–N * 44/28 (IPCC, 2006) and converted to kg of CO_2_-eq on the basis of 100-years GWP of 298. Hence, for every kg of N fertilizer, GWP from indirect N_2_O emissions is 0.46 and 1.04 kg CO_2_-eq from volatilization and leaching losses, respectively. Last, total CO_2_-eq were divided as a function of the total surface required for the output in the blending scenario.

## Results

### Grain yield differences between crop types

**Figure 2:**
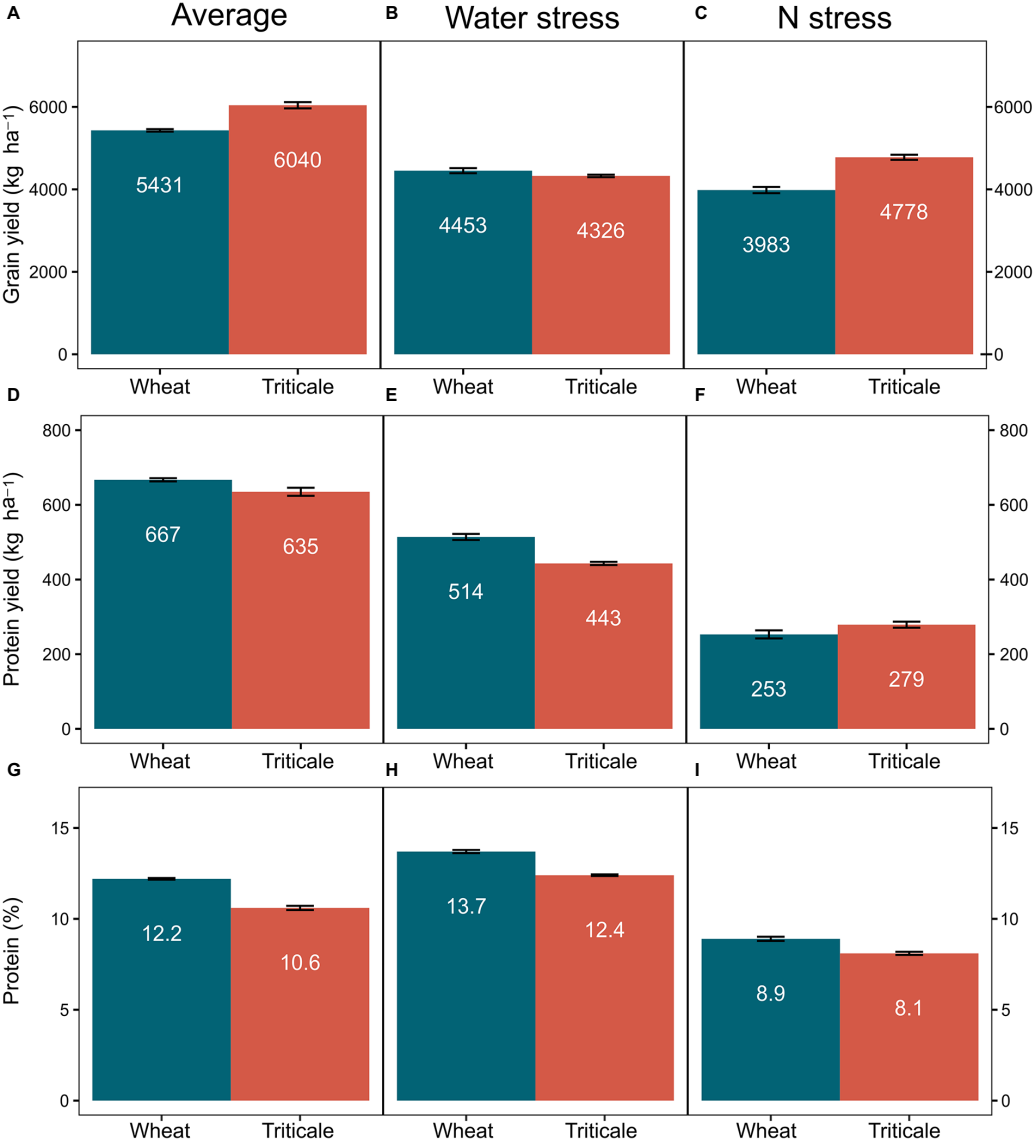
Estimated marginal means (± SE) from models in Table 2 for grain yield **(A–C)**, protein yield **(D–F)**, and protein concentration **(G–I)** for common wheat and triticale growing under average conditions, water and N stressed environments. Numbers in white are the values for each bar.

Differences between crop types were significant for grain yield, protein yield, and grain protein concentration (Table 2). All response variables resulted in significant interactions with water-and N-stress conditions. Triticale outyielded wheat under average and N stressed conditions with a mean difference of 606 and 795 kg ha^−1^, respectively (*p* < 0.05). Whereas triticale was 127 kg ha^−1^ lower yielding than wheat (*p* < 0.05) under water stressed environments (Figure 2B).

**Table 2:** Analysis of variance for grain yield, protein yield, and protein concentration.

	Yield	Protein yield	Protein (%)
Crop type	<0.001	<0.001	<0.001
Water stress	0.170	0.010	0.007
N stress	0.013	<0.001	<0.001
Crop type × Water stress	<0.001	<0.001	0.03
Crop type × N stress	0.002	<0.001	<0.001

Protein concentration was significantly lower in triticale in all conditions (Table 2; Figure 2). However, given the higher yields of triticale, protein yield differences were smaller. For instance, for the average conditions, wheat protein concentration was 15% higher than triticale whereas protein yield was only 5% greater. Likewise, under N-stress conditions the ranking was inverted for protein yield, with 10% higher protein yield for triticale despite protein concentration being lower. Under water-stress conditions, rankings for protein concentration and protein yield were consistent due to similar grain yields among the two crop types.

### Differences in grain yield stability between wheat and triticale

**Figure 3:**
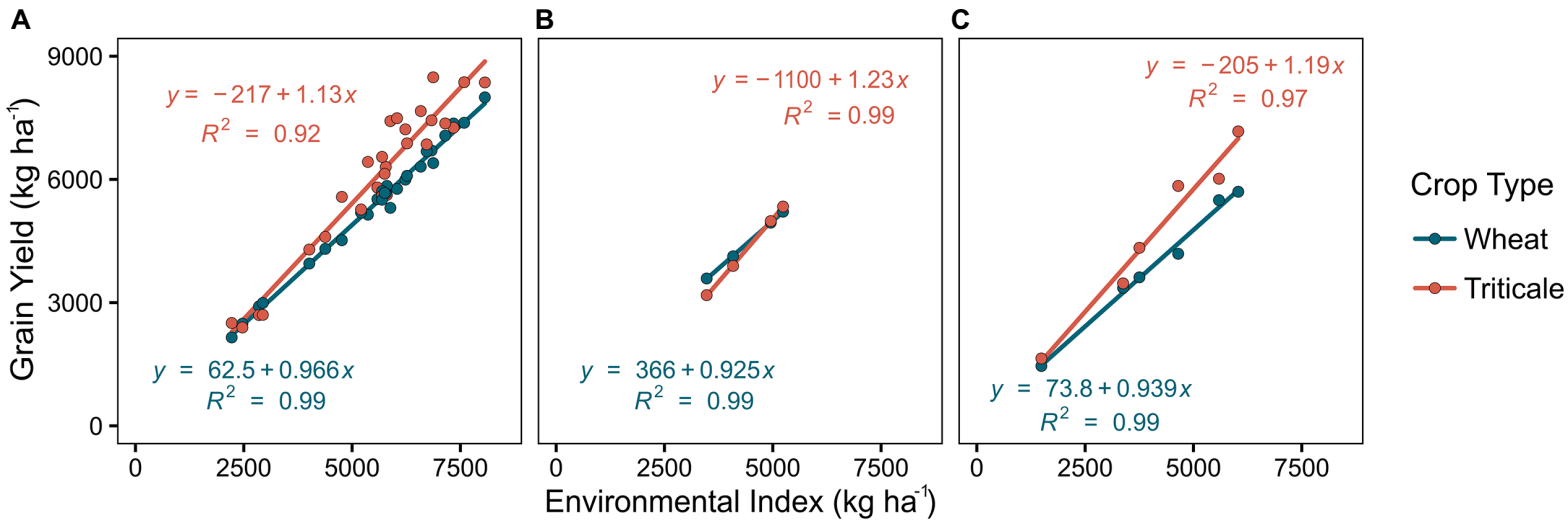
Relationship between yield and environmental index (EI) for wheat and triticale crop types under average growing conditions **(A)**, water-stress conditions **(B)**, and N-stress conditions **(C)**. The environmental index is the average yield of all varieties tested each site-year.

We tested differences between crop types in their yield response across the different growing conditions following an environmental index approach (Figure 3). Similar to trends observed for average conditions, triticale performed equal or better than wheat across all environmental gradients under average and N-stress conditions (Figures 3A,C), showing a similar response to wheat in low-yielding environments but a greater response in medium and high-yielding environments. The steeper slope for triticale indicated that for every increase in one unit of EI, triticale grain yield was 17 and 16% higher than wheat (Figures 3A,C) under average conditions and N-stress conditions, respectively. In contrast, differences are less pronounced when water is the primary limitation to crop growth and maximum yields remain under 5,237 kg ha^−1^ (Figure 3B). Further, wheat has higher productivity under these conditions for protein concentration and yield (Figures 2B,H).

### Water productivity and PFPN between crop types and implications for flour blending

**Figure 4:**
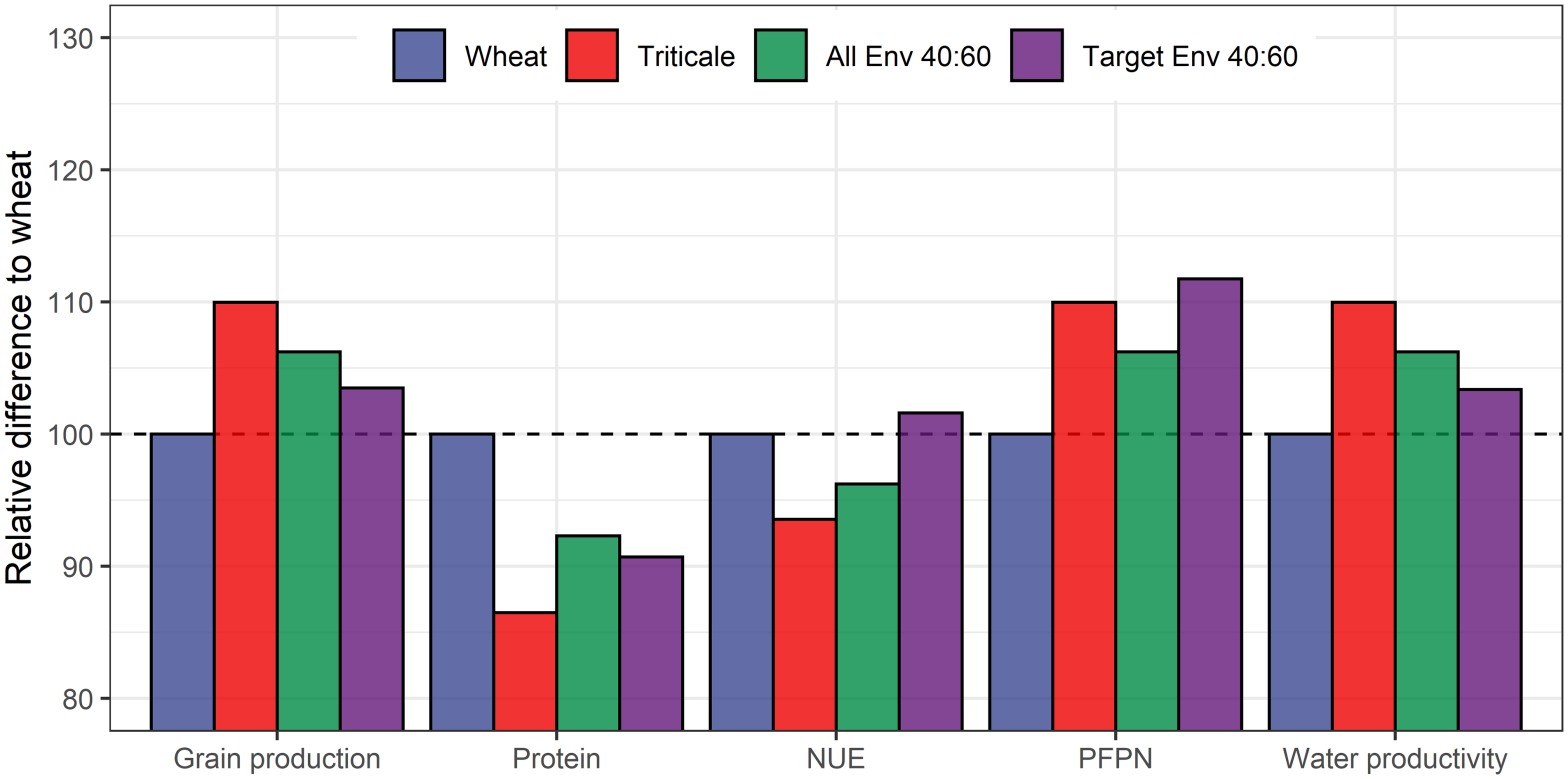
Graphical representation of relative values in Table 3 comparing grain production, protein concentration, and productivity metrics to a baseline of 100% wheat (blue bars) or triticale (red bars) production compared to a 40:60 (green bars) and 40:60 (purple bars) for all and targeted environments, respectively.

To represent the implications of these differences, we calculated different scenarios based on the multi-environment results from the entire dataset (Table 3). If wheat and triticale were planted across the entire range of environments in this study and their resulting grains were used to create flour in a blending ratio of 40:60 (wheat to triticale), estimates indicate that total grain production, water productivity and PFPN would be 6% higher than the baseline of producing only wheat in these same environments (Figure 4).

**Table 3:** Grain production, protein concentration, nitrogen use efficiency (NUE), partial factor productivity of N (PFPN) and water productivity for wheat, triticale, and two scenarios of flour blending (40:60).

Variable	Wheat	Triticale	All environments	Target environments
	**100%**	**100%**	**40:60**	**40:60**
Grain production (kg ha^−1^)	5,090	5,653	5,427	5,273
Protein concentration (%)	11.8	10.4	11.0	10.8
NUE	0.80	0.75	0.77	0.82
PFPN (kg kg N^−1^)	38.4	42.7	41.0	43.6
Water productivity (kg mm^−1^)	9.0	10.0	9.6	9.3

One scenario where crop types are not targeted to specific environments and another one where wheat is cultivated in low-yielding environments (EI < 4,522 kg ha^−1^) and triticale in high environments (EI > 4,522 kg ha^−1^).

On the other hand, protein concentration and NUE would decrease by 8 and 4%, respectively. By comparison, if wheat and triticale production were targeted to environments where each has resource use efficiency advantages and their flour were combined in the same 40:60 ratio, NUE would increase by 2% while maintaining a higher PFPN (12%), water productivity (3%), and grain production (3%) above the wheat-only baseline. Yet, this approach would represent a decrease of 9% for protein concentration. However, higher overall grain production would compensate for these differences in grain quality. Likewise, the higher productivity of triticale in the blending would not compromise NUE, which would be 2% higher under this scenario.

The strategy of targeting optimum environments for each crop type would translate to reduced agronomic inputs based on differences in PFPN and the resulting N fertilizer savings and associated implications for GWP estimates. Specifically, a 40:60 blend of flour sourced from targeted environments compared to the current scenario of only producing wheat flour could represent a regional-scale savings in fertilizer of 555 Mg N based on differences in PFPN (Table 4). Likewise, the total reduction of GWP derived from reduction in N fertilizer would be 166 kg of CO_2_-eq ha^−1^ as a result of strategically sourced and blended flour (Table 4).

**Table 4:** Differences between production scenarios comparing 100% wheat and 40:60 blending (wheat:triticale) from targeted environments and its implications for fertilizer N savings and GWP expressed in CO_2_-eq based on differences in improved PFPN.

	100% Wheat	Blend 40:60
PFPN (kg kg N^−1^)	38.4	43.6
Wheat produced in California (kg)	178,563,200	178,563,200
Surface required in each scenario (ha)	35,081	33,864
N fertilizer required in each scenario (kg)	4,650,083	4,095,486
**Total savings from improved PFPN**		
N saved (kg)		554,597
CO_2_-eq (kg)		5,629,161
CO_2_-eq (kg ha^−1^)		166
CO_2_-eq Mg grain^−1^		32

## Discussion

Triticale outyielded wheat by 11% on average (Figure 2) under normal growing conditions across a wide range of commercial varieties and environments representative of the California wheat production area. Compared to triticale, wheat had a lower capacity to tolerate N stress under the conditions explored in this study, but had better performance under water stress. Agronomically, these results indicate that wheat and triticale production could be targeted to different environments to increase total production while being more efficient in the utilization of water and N fertilizer inputs, two key factors underpinning the sustainability of cereal cropping systems (Fischer and Connor, 2018; Cassman and Grassini, 2020). Specifically, wheat production would be best targeted in areas that are primarily water-limited and low-yielding, whereas triticale production would be best targeted in areas that are typically N-limited and high-yielding (e.g., irrigated fields). This approach would result in an overall increase in PFPN and water productivity (Figure 4). California is facing new regulations for water and N inputs and these results provide novel mechanisms and approaches for maintaining agronomic productivity while reducing freshwater resource consumption, nutrient pollution, and GHG emissions.

Our results agree with previous studies reporting yield superiority of triticale, yet the broad number of varieties and environments reported here is unique in the literature. Higher yield advantage of triticale has been reported in other wheat production regions in the United Kingdom (Roques et al., 2017), Europe (Giunta et al., 1993; Villegas et al., 2010), the US (Schillinger and Archer, 2020), Australia (López-Castañeda and Richards, 1994a) and South America (Estrada-Campuzano et al., 2012). In other Mediterranean climates, similar trends were found for grain yield in response to EI, depicting a higher adaptability and yield potential in triticale than wheat (Josephides, 1992; Méndez-Espinoza et al., 2019). Previous studies analyzing physiological traits in triticale, have attributed yield advantages to higher aboveground biomass accumulation associated with higher radiation use efficiency (Giunta et al., 2009; Estrada-Campuzano et al., 2012) and greater photosynthetic and carbon assimilation rates during the grain filling (Méndez-Espinoza et al., 2019). Even though we did not perform specific measurements, the slightly lower performance of triticale in the water stressed condition could be related to vigorous vegetative growth (López-Castañeda and Richards, 1994b) and a larger root system (Richards et al., 2007) in early developmental stages compared to wheat. These traits might have resulted in greater depletion of initial soil water availability, compromising post-anthesis growth, particularly in the fields classified as low-yielding that received an average of 143 mm less water than the high-yielding cluster. The better performance of wheat when late season water is scarce has been confirmed before in extensive trials covering broad range of environments (Josephides, 1992; Reynolds et al., 2002). Thus, it is likely that a similar dynamic contributed to minimize the differences between the two crops at low water inputs sites (e.g., low end of EI response curves).

Our results showed higher average protein concentration in wheat under all treatments regardless yield differences with triticale. Increases in grain protein concentration without changes in grain yield is commonly observed in wheat N fertilization trials (Walsh et al., 2020) with negative implications for NUE (Walsh et al., 2022). Conceptually, increasing yield and NUE in wheat would only be achieved at the expense of grain protein concentration (Barraclough et al., 2010) which is not desirable for the baking industry. On the other hand, triticale produced more grain per unit of water and N fertilizer inputs at the expense of lower protein concentration and NUE. Nevertheless, we demonstrate that the tradeoff with NUE can be resolved by targeting specific environments that maximize the advantages of each crop type. In all scenarios, including triticale improved PFPN, water productivity and grain production despite that all combinations led to lower protein concentrations compared with the wheat baseline (Table 3; Figure 4). The targeted 40:60 scenario, brings a balanced outcome for all metrics despite a small overall reduction in grain yield and water productivity due to the portion of grain sourced from wheat produced in low-yielding environments (Figure 1).

The exploratory nature of our analysis implies that fine-tuning existing management practices reported in other production systems and climates may offer even greater enhancements in sustainability and productivity metrics. For instance, in humid continental regions of the US, shifting planting dates earlier in the season has been shown to increase early-season uptake of residual N and crop productivity of triticale while decreasing N losses from the soil system (Nance et al., 2007; Lyons et al., 2018). Likewise, selection of varieties to avoid plant lodging (Yang et al., 2022), diseases (Rodriguez-Algaba et al., 2019) or feed quality issues (Giunta et al., 2020) are important aspects to consider in triticale systems. However, further research would be necessary to confirm the benefits of these production practices in California environments and to implement or adapt them in those settings.

These results have several implications for the regional N economy. Specifically, (1) the risk of N losses can be reduced through increased net grain N recovery and export from fields, and (2) the overall consumption of N fertilizers can be decreased, translating to lower GHG emissions from the cropping system. Improving NUE is key to decreasing the risk of N losses while maintaining high crop productivity (Cassman and Dobermann, 2021). Accordingly, NUE is an important indicator for assessing the sustainability of cropping systems (Zhang et al., 2015). Most efforts to improve NUE focuses on the implementation of better N management practices at the field-or farm-scale (e.g., seeking to match soil N availability with N crop demand without incurring yield penalties). Here we demonstrate that the strategic co-production of triticale and wheat can also increase overall NUE, suggesting this would be an additional method for reducing N consumption in grain production for the baking industry. While this strategy reflects the efficiency achieved at the cultivation phase, progress toward reducing the environmental footprint should target the agri-food chain as a whole through a coordinated effort from the parts involved (Horton, 2017). For instance, better awareness of the tradeoffs in N use among actors throughout the supply chain is needed. Studies on nutrient management in other systems have demonstrated that N losses in subsequent production phases (e.g., feed conversion in dairy systems) can lead to lower overall N efficiencies in the food chain (Martínez-Blanco et al., 2011; Powell and Rotz, 2015) despite achieving higher efficiency in the cultivation phase.

Prospects for triticale as an alternative crop to mitigate GHG emissions are favorable due to its versatility for other end uses. Triticale grain can be used to make biomaterials, biocomponents and energy (Dassanayake and Kumar, 2012; Sanaei and Stuart, 2018) and has demonstrated advantages in other sectors of the ag-related industry. For instance, higher net benefits of reducing the C footprint in bioethanol production are obtained when using triticale instead of wheat due to its lower N fertilizer requirements (Weightman and Davis-Knight, 2008). Moreover, the adoption of specific complementary farming practices that were not addressed in this study could further contribute to GHG mitigation without impacting yields. Our estimate of 166 kg CO_2_ eq ha^−1^ was calculated only based on differences in PFPN between crop types. In other studies, wheat systems in semiarid environments lower their C footprint by an average of 256 kg CO_2_ eq ha^−1^ per year when combining the effects of optimized fertilization, reduced summer fallow frequencies, and the inclusion of legumes in the crop rotation (Gan et al., 2014). In addition, wheat systems in the United Kingdom reduced GHG emissions by 15% by utilizing fungicides and increasing yields (Berry et al., 2008). Further, in Mediterranean regions, triticale systems reduced methane and N_2_O emissions when rotated with legumes (Oliveira et al., 2021). These examples argue for additional study of potential agronomic advantages and synergies associated with the intensification of triticale in the agri-food chain.

Human dietary habits can play an important role in mitigating GHG emissions (Lassaletta et al., 2014). In addition to environmental impacts, dietary changes might offer positive impacts on human health. In this instance, expanded use of triticale in the breadmaking industry has the potential to enhance human health based on its nutritional properties (Mosse et al., 1988; Onwulata et al., 2000). However, a major challenge for triticale adoption is the requirement to mix with some proportion of wheat flour to achieve acceptable baking results. The ratio between flours has been a recurrent topic in the scientific literature, and a number of studies agree that acceptable dough can be achieved by blending wheat flour with 50 to 60% triticale flour (Naeem et al., 2002; Serna-Saldivar et al., 2004; Coskuner and Karababa, 2005; Birou et al., 2010).

We recognize that economic analysis is an important limitation of our study, especially considering input and irrigation costs as well as crop prices for wheat and triticale under different market conditions. Because the price of triticale is generally lower than wheat at present, the adoption of triticale in wheat producing regions could be challenging even after considering yield advantages for most of the production sites. Despite these uncertainties, emerging environmental policies and other economic instruments supporting more efficient use of inputs could potentially offset these differences. For instance, water markets (Ayres et al., 2021) and C markets promoting better use of N fertilizer (CAR, 2022) are some programs currently in place in California. Likewise, consumer-oriented policies (e.g., price controls) could contribute to expanding domestic markets in favor of more sustainable products. In addition, efforts are increasing from the private sector to raise awareness and reduce environmental footprint in agri-food chains by supporting sustainable sourcing (Gillum et al., 2016; Borsellino et al., 2020) or implementing environmental indicators to track N pollution in the food-chain (McLellan et al., 2018; Tamagno et al., 2022). Therefore, while triticale baking products have yet to develop a large-scale niche within the agri-food chain, increasing public awareness of food production externalities may present an opportunity for increased utilization of triticale in food products.

## Conclusion

Our results support the hypothesis that increasing the proportion of triticale in baking supply chains could decrease the environmental footprint of resulting food products. Triticale produced higher yields and yield stability under a wide range of growing conditions, including N-limited environments, while wheat performance was superior under water-limited conditions. These results suggest that, in order to optimize the agronomic and environmental benefits, cultivation of triticale should be strategically targeted to high-yielding environments and wheat to low-yielding environments to support an overall blending ratio of 40:60 for the milling industry. In this way, total grain production would increase relative to the current wheat-only baseline while having a synergistic effect for selected metrics related to N inputs and water use, and maintaining acceptable NUE. Considering future needs in the region to increase resource use efficiency and reduce GHG emissions, these results present novel approaches for maintaining productivity while improving system sustainability. Beyond this conclusion, this study illustrates the relatively underexplored benefits that might be derived from coordinating complementarities among crops at multiple links in the agri-food chain. In this instance, coordinating the blending of flour with the targeted cultivation of two crops based on favorable crop production conditions would result in a positive environmental impact. Therefore, this study also demonstrates the potential for leveraging species-environment interactions to benefit overall resource use efficiency and system sustainability at the regional-scale.

## Data availability statement

The original contributions presented in the study are publicly available. This data can be found at the University of California Small Grains Research and Information Center Website (https://smallgrains.ucanr.edu/Variety_Selection/).

## Author contributions

ST: conceptualization, data curation, formal analysis, investigation, methodology, visualization, writing—original draft, and writing—review and editing. CP: conceptualization, methodology, and writing—review and editing. GF, JH, CC, and TV: writing—review editing. TN: data curation and writing—review editing. ML: conceptualization, resources, funding acquisition, methods, data curation, writing—review editing, and supervision. All authors contributed to the article and approved the submitted version.

## Conflict of interest

The authors declare that the research was conducted in the absence of any commercial or financial relationships that could be construed as a potential conflict of interest.

## Publisher’s note

All claims expressed in this article are solely those of the authors and do not necessarily represent those of their affiliated organizations, or those of the publisher, the editors and the reviewers. Any product that may be evaluated in this article, or claim that may be made by its manufacturer, is not guaranteed or endorsed by the publisher.

## References

[ref1] Ayres A. Hanak E. Gray B. Sencan G. Bruno E. Escriva-Bou A.. (2021). Improving California’s Water Market: How Water Trading and Banking Can Support Groundwater Management. Public Policy Institute of California. Available at: https://www.ppic.org/publication/improving-californias-water-market/

[ref2] Bakhshi A. K. Sehgal K. L. Pal Singh R. Gill K. S. (1989). Effect of bread wheat, durum wheat and triticale blends on Chapati, bread and biscuit. J. food sci. tech. (Mysore) 26, 191–193.

[ref3] Barraclough P. B. Howarth J. R. Jones J. Lopez-Bellido R. Parmar S. Shepherd C. E.. (2010). Nitrogen efficiency of wheat: genotypic and environmental variation and prospects for improvement. Eur. J. Agron. 33, 1–11. doi: 10.1016/j.eja.2010.01.005

[ref4] Bates D. Mächler M. Bolker B. Walker S. (2015). Fitting linear mixed-effects models using lme4. J. Stat. Softw. 67:01. doi: 10.18637/jss.v067.i01

[ref5] Benaglia T. Chauveau D. Hunter D. R. Young D. S. (2010). Mixtools: an R package for analyzing mixture models. J. Stat. Softw. 32, 1–29. doi: 10.18637/jss.v032.i06

[ref6] Berry P. M. Kindred D. R. Paveley N. D. (2008). Quantifying the effects of fungicides and disease resistance on greenhouse gas emissions associated with wheat production. Plant Pathol. 57, 1000–1008. doi: 10.1111/j.1365-3059.2008.01899.x

[ref7] Birou A. Muste S. Man S. Muresan V. Chircu C. Kadar R. (2010). “Optimizing the wheat/triticale ratio to improve the quality parameters of bakery products,” in Bulletin of the University of Agricultural Sciences & Veterinary Medicine Cluj-Napoca. Agriculture, Vol. 67.

[ref8] Blum A. (2014). The abiotic stress response and adaptation of triticale — A review. Cereal Res. Commun. 42, 359–375. doi: 10.1556/CRC.42.2014.3.1

[ref9] Borsellino V. Schimmenti E. El Bilali H. (2020). Agri-food markets towards sustainable patterns. Sustain. For. 12:2193. doi: 10.3390/su12062193

[ref10] Calviño P. A. Sadras V. O. (2002). On-farm assessment of constraints to wheat yield in the south-eastern pampas. Field Crop Res. 74, 1–11. doi: 10.1016/S0378-4290(01)00193-9

[ref11] CAR (2022). Register a compliance offset project. Available at: https://www.climateactionreserve.org/how/california-compliance-projects/register-a-compliance-offset-project/ (Accessed April 5, 2022).

[ref12] Cassman K. G. Dobermann A. R. (2021). Nitrogen and the future of agriculture: 20 years on. Ambio 51, 17–24. doi: 10.1007/s13280-021-01526-w, PMID: 33715091 PMC8651835

[ref13] Cassman K. G. Grassini P. (2020). A global perspective on sustainable intensification research. Nature Sustain. 3, 262–268. doi: 10.1038/s41893-020-0507-8

[ref14] Congreves K. A. Otchere O. Ferland D. Farzadfar S. Williams S. Arcand M. M. (2021). Nitrogen use efficiency definitions of today and tomorrow. Front. Plant Sci. 12:637108. doi: 10.3389/fpls.2021.637108, PMID: 34177975 PMC8220819

[ref15] Coskuner Y. Karababa E. (2005). Studies on the quality of Turkish flat breads based on blends of triticale and wheat flour. Int. J. Food Sci. Technol. 40, 469–479. doi: 10.1111/j.1365-2621.2005.00925.x

[ref16] Cossani C. M. Sadras V. O. (2018). Water–nitrogen Colimitation in grain crops. Adv. Agron. 150, 231–274. doi: 10.1016/bs.agron.2018.02.004

[ref17] Cossani C. M. Slafer G. A. Savin R. (2010). Co-limitation of nitrogen and water, and yield and resource-use efficiencies of wheat and barley. Crop Pasture Sci. 61:844. doi: 10.1071/CP10018

[ref18] Dassanayake G. D. M. Kumar A. (2012). Techno-economic assessment of triticale straw for power generation. Appl. Energy 98, 236–245. doi: 10.1016/j.apenergy.2012.03.030

[ref19] Deng L. Zhang H. Wang C. Ma W. Zhu A. Zhang F.. (2021). Improving the sustainability of the wheat supply chain through multi-stakeholder engagement. J. Clean. Prod. 321:128837. doi: 10.1016/j.jclepro.2021.128837, PMID: 34720459 PMC8527860

[ref20] Dettinger M. D. Ralph F. M. Das T. Neiman P. J. Cayan D. R. (2011). Atmospheric Rivers, floods and the water resources of California. WaterSA 3, 445–478. doi: 10.3390/w3020445

[ref21] Ellen J. (1993). Growth, yield and composition of four winter cereals. I. Biomass, grain yield and yield formation. Neth. J. Agric. Sci. 41, 153–165. doi: 10.18174/njas.v41i2.628

[ref22] Estrada-Campuzano G. Slafer G. A. Miralles D. J. (2012). Differences in yield, biomass and their components between triticale and wheat grown under contrasting water and nitrogen environments. Field Crop Res. 128, 167–179. doi: 10.1016/j.fcr.2012.01.003

[ref23] FAO (2010). AQUASTAT-FAO’s global information system on water and agriculture. Available at: https://www.fao.org/aquastat/en/overview/methodology/water-use (Accessed December 6, 2021).

[ref24] FAOSTAT (2021). Food and agriculture Organization of the United Nations. Available at: *www.fao.org*

[ref25] Fernández J. E. Alcon F. Diaz-Espejo A. Hernandez-Santana V. Cuevas M. V. (2020). Water use indicators and economic analysis for on-farm irrigation decision: A case study of a super high density olive tree orchard. Agric. Water Manag. 237:106074. doi: 10.1016/j.agwat.2020.106074

[ref26] Finlay K. W. Wilkinson G. N. (1963). The analysis of adaptation in a plant-breeding programme. Aust. J. Agric. Res. 14:742. doi: 10.1071/AR9630742

[ref27] Fischer R. A. Connor D. J. (2018). Issues for cropping and agricultural science in the next 20 years. Field Crop Res. 222, 121–142. doi: 10.1016/j.fcr.2018.03.008

[ref28] Fowler D. Coyle M. Skiba U. Sutton M. A. Cape J. N. Reis S.. (2013). The global nitrogen cycle in the twenty-first century. Philosop. Trans. Royal Soc. B: Biolog. Sci. 368:20130164. doi: 10.1098/rstb.2013.0164, PMID: 23713126 PMC3682748

[ref29] Fujihara S. Sasaki H. Aoyagi Y. Sugahara T. (2008). Nitrogen-to-protein conversion factors for Some cereal products in Japan. J. Food Sci. 73, C204–C209. doi: 10.1111/j.1750-3841.2008.00665.x, PMID: 18387100

[ref30] Gan Y. Liang C. Chai Q. Lemke R. L. Campbell C. A. Zentner R. P. (2014). Improving farming practices reduces the carbon footprint of spring wheat production. Nat. Commun. 5:5012. doi: 10.1038/ncomms6012, PMID: 25405548 PMC4243251

[ref31] Geisseler D. (2016). Nitrogen Concentrations in Harvested plant Parts - A Literature Overview. California: University of California, 157.

[ref32] Gillum M. Johnson P. Hudson D. Williams R. (2016). Fieldprint calculator: A tool to evaluate the effects of management on physical sustainability. Crops. Soils 49, 26–29. doi: 10.2134/cs2016-49-1-7

[ref33] Giunta F. Cadeddu F. Mureddu F. Virdis A. Motzo R. (2020). Triticale cultivar mixtures: productivity, resource use and resource use efficiency in a Mediterranean environment. Eur. J. Agron. 115:126019. doi: 10.1016/j.eja.2020.126019

[ref34] Giunta F. Motzo R. Deidda M. (1993). Effect of drought on yield and yield components of durum wheat and triticale in a Mediterranean environment. Field Crop Res. 33, 399–409. doi: 10.1016/0378-4290(93)90161-F

[ref35] Giunta F. Pruneddu G. Motzo R. (2009). Radiation interception and biomass and nitrogen accumulation in different cereal and grain legume species. Field Crop Res. 110, 76–84. doi: 10.1016/j.fcr.2008.07.003

[ref36] Goucher L. Bruce R. Cameron D. D. Lenny Koh S. C. Horton P. (2017). The environmental impact of fertilizer embodied in a wheat-to-bread supply chain. Nature Plants 3:17012. doi: 10.1038/nplants.2017.12 28248299

[ref37] Gutteridge R. J. Hornby D. Hollins T. W. Prew R. D. (1993). Take-all in autumn-sown wheat, barley, triticale and rye grown with high and low inputs. Plant Pathol. 42, 425–431. doi: 10.1111/j.1365-3059.1993.tb01521.x

[ref38] Horton P. (2017). We need radical change in how we produce and consume food. Food Security 9, 1323–1327. doi: 10.1007/s12571-017-0740-9

[ref39] IPCC (2006). Intergovernmental Panel on Climate Change Guidelines for National Greenhouse Gas Inventories. Volume 4: Agriculture, Forestry and Other Land Use. Chapter 11: N2O Emissions from Managed Soils, and CO2 Emissions from Lime and Urea Application. Available at: http://www.ipcc-nggip.iges.or.jp/public/2006gl/ pdf/4_Volume4/V4_11_Ch11_N2O&CO2.pdf

[ref40] Josephides C. M. (1992). Analysis of adaptation of barley, triticale, durum and bread wheat under Mediterranean conditions. Euphytica 65, 1–8. doi: 10.1007/BF00022193

[ref41] Ladha J. K. Pathak H. J. Krupnik T. Six J. van Kessel C. (2005). Efficiency of fertilizer nitrogen in cereal production: Retrospects and prospects. Adv. Agron. 87, 85–156. doi: 10.1016/S0065-2113(05)87003-8

[ref42] Lassaletta L. Billen G. Grizzetti B. Anglade J. Garnier J. (2014). 50 year trends in nitrogen use efficiency of world cropping systems: the relationship between yield and nitrogen input to cropland. Environ. Res. Lett. 9:105011. doi: 10.1088/1748-9326/9/10/105011

[ref43] López-Castañeda C. Richards R. A. (1994a). Variation in temperate cereals in rainfed environments I. grain yield, biomass and agronomic characteristics. Field Crop Res. 37, 51–62. doi: 10.1016/0378-4290(94)90081-7

[ref44] López-Castañeda C. Richards R. A. (1994b). Variation in temperate cereals in rainfed environments II. Phasic development and growth. Field Crop Res. 37, 63–75. doi: 10.1016/0378-4290(94)90082-5

[ref45] Lyons S. E. Ketterings Q. M. Godwin G. Cherney J. H. Czymmek K. J. Kilcer T. (2018). Spring nitrogen management is important for triticale forage yield and quality. Agron. J. 110, 2025–2032. doi: 10.2134/agronj2018.01.0041

[ref47] Martínez-Blanco J. Muñoz P. Antón A. Rieradevall J. (2011). Assessment of tomato Mediterranean production in open-field and standard multi-tunnel greenhouse, with compost or mineral fertilizers, from an agricultural and environmental standpoint. J. Clean. Prod. 19, 985–997. doi: 10.1016/j.jclepro.2010.11.018

[ref48] McLellan E. L. Cassman K. G. Eagle A. J. Woodbury P. B. Sela S. Tonitto C.. (2018). The nitrogen balancing act: tracking the environmental performance of food production. Bioscience 68, 194–203. doi: 10.1093/biosci/bix164, PMID: 29662247 PMC5894078

[ref49] Méndez-Espinoza A. M. Romero-Bravo S. Estrada F. Garriga M. Lobos G. A. Castillo D.. (2019). Exploring agronomic and physiological traits associated With the differences in productivity Between triticale and bread wheat in Mediterranean environments. Front. Plant Sci. 10:404. doi: 10.3389/fpls.2019.00404, PMID: 31024582 PMC6460938

[ref50] Mergoum M. Pfeiffer W. H. Peña R. J. Ammar K. Rajaram S. (2004). Triticale crop improvement: the CIMMYT programme. Triticale improv. product. 179, 11–26.

[ref51] Mosse J. Huet J. C. Baudet J. (1988). The amino acid composition of triticale grain as a function of nitrogen content: comparison with wheat and rye. J. Cereal Sci. 7, 49–60. doi: 10.1016/S0733-5210(88)80059-6

[ref52] Naeem H. A. Darvey N. L. Gras P. W. MacRitchie F. (2002). Mixing properties, baking potential, and functionality changes in storage proteins During dough development of triticale-wheat flour blends. Cereal Chem. J. 79, 332–339. doi: 10.1094/CCHEM.2002.79.3.332

[ref53] Nance C. D. Gibson L. R. Karlen D. L. (2007). Soil profile nitrate response to nitrogen fertilization of winter triticale. Soil Sci. Soc. Am. J. 71, 1343–1351. doi: 10.2136/sssaj2006.0262

[ref54] NASS-USDA (2022). 2021 State Agriculture Overview. Available at: https://www.nass.usda.gov/Quick_Stats/Ag_Overview/stateOverview.php?state=CALIFORNIA

[ref55] Oliveira M. Castro C. Coutinho J. Trindade H. (2021). Grain legume-based cropping systems can mitigate greenhouse gas emissions from cereal under Mediterranean conditions. Agric. Ecosyst. Environ. 313:107406. doi: 10.1016/j.agee.2021.107406

[ref56] Onwulata C. I. Konstance R. P. Strange E. D. Smith P. W. Holsinger V. H. (2000). High-fiber snacks extruded from triticale and wheat formulations. Cereal Foods World 45, 470–473.

[ref57] Pathak T. Maskey M. Dahlberg J. Kearns F. Bali K. Zaccaria D. (2018). Climate change trends and impacts on California agriculture: A detailed review. Agronomy 8:25. doi: 10.3390/agronomy8030025

[ref58] Powell J. M. Rotz C. A. (2015). Measures of nitrogen use efficiency and nitrogen loss from dairy production systems. J. Environ. Qual. 44, 336–344. doi: 10.2134/jeq2014.07.0299, PMID: 26023953

[ref59] Reynolds M. P. Trethowan R. Crossa J. Vargas M. Sayre K. D. (2002). Physiological factors associated with genotype by environment interaction in wheat. Field Crop Res. 75, 139–160. doi: 10.1016/S0378-4290(02)00023-0

[ref60] Richards R. A. Watt M. Rebetzke G. J. (2007). Physiological traits and cereal germplasm for sustainable agricultural systems. Euphytica 154, 409–425. doi: 10.1007/s10681-006-9286-1

[ref61] Rodrigues G. C. Pereira L. S. (2009). Assessing economic impacts of deficit irrigation as related to water productivity and water costs. Biosyst. Eng. 103, 536–551. doi: 10.1016/j.biosystemseng.2009.05.002

[ref62] Rodriguez-Algaba J. Sørensen C. K. Labouriau R. Justesen A. F. Hovmøller M. S. (2019). Susceptibility of winter wheat and triticale to yellow rust influenced by complex interactions between Vernalisation, temperature, plant growth stage and pathogen race. Agronomy 10:13. doi: 10.3390/agronomy10010013

[ref63] Roques S. E. Kindred D. R. Clarke S. (2017). Triticale out-performs wheat on range of UK soils with a similar nitrogen requirement. J. Agric. Sci. 155, 261–281. doi: 10.1017/S0021859616000356

[ref64] Sadras V. O. Hayman P. T. Rodriguez D. Monjardino M. Bielich M. Unkovich M. J.. (2016). Interactions between water and nitrogen in Australian cropping systems: physiological, agronomic, economic, breeding and modelling perspectives. Crop Pasture Sci. 67, 1019–1053. doi: 10.1071/CP16027

[ref65] Salmeron M. Gbur E. E. Bourland F. M. Buehring N. W. Earnest L. Fritschi F. B.. (2014). Soybean maturity group choices for early and late plantings in the Midsouth. Agron. J. 106, 1893–1901. doi: 10.2134/agronj14.0222

[ref66] Sanaei S. Stuart P. R. (2018). Systematic assessment of triticale-based biorefinery strategies: techno-economic analysis to identify investment opportunities. Biofuels Bioprod. Biorefin. 12, S46–S59. doi: 10.1002/bbb.1499

[ref67] Schillinger W. F. Archer D. W. (2020). Winter triticale: A long-term cropping systems experiment in a dry Mediterranean climate. Agronomy 10:1777. doi: 10.3390/agronomy10111777

[ref68] Serna-Saldivar S. O. Guajardo-Flores S. Viesca-Rios R. (2004). Potential of triticale as a substitute for wheat in flour tortilla production. Cereal Chem. J. 81, 220–225. doi: 10.1094/CCHEM.2004.81.2.220

[ref69] Shewry P. R. Hey S. J. (2015). The contribution of wheat to human diet and health. Food. Energy Security 4, 178–202. doi: 10.1002/fes3.64, PMID: 27610232 PMC4998136

[ref70] Sinha S. K. Aggarwal P. K. Chaturvedi G. S. Singh A. K. Kailasnathan K. (1986). Performance of wheat and triticale cultivars in a variable soil—water environment I. grain yield stability. Field Crop Res. 13, 289–299. doi: 10.1016/0378-4290(86)90031-6

[ref71] Snyder C. S. Bruulsema T. W. Jensen T. L. Fixen P. E. (2009). Review of greenhouse gas emissions from crop production systems and fertilizer management effects. Agric. Ecosyst. Environ. 133, 247–266. doi: 10.1016/j.agee.2009.04.021

[ref72] Springmann M. Clark M. Mason-D’Croz D. Wiebe K. Bodirsky B. L. Lassaletta L.. (2018). Options for keeping the food system within environmental limits. Nature 562, 519–525. doi: 10.1038/s41586-018-0594-0, PMID: 30305731

[ref73] Tamagno S. Eagle A. J. McLellan E. L. van Kessel C. Linquist B. A. Ladha J. K.. (2022). Quantifying N leaching losses as a function of N balance: A path to sustainable food supply chains. Agric. Ecosyst. Environ. 324:107714. doi: 10.1016/j.agee.2021.107714

[ref74] Tamagno S. Greco I. A. Almeida H. Paola J. C. Di. (2016). Crop management options for maximizing maize kernel hardness. Agron. J. 108, 1561–1570. doi:10.2134/agronj2015.0590

[ref75] van Herwaarden A. F. Farquhar G. D. Angus J. F. Richards R. A. Howe G. N. (1998). “Haying-off”, the negative grain yield response of dryland wheat to nitrogen fertiliser. I. Biomass, grain yield, and water use. Aust. J. Agric. Res. 49:1067. doi: 10.1071/A97039

[ref76] Vermeulen S. J. Campbell B. M. Ingram J. S. I. (2012). Climate change and food systems. Annu. Rev. Environ. Resour. 37, 195–222. doi: 10.1146/annurev-environ-020411-130608

[ref77] Villegas D. Casadesús J. Atienza S. G. Martos V. Maalouf F. Karam F.. (2010). Tritordeum, wheat and triticale yield components under multi-local mediterranean drought conditions. Field Crop Res. 116, 68–74. doi: 10.1016/j.fcr.2009.11.012

[ref78] Walsh O. S. Marshall J. Nambi E. Shafian S. Jayawardena D. Jackson C.. (2022). Spring wheat yield and grain quality response to nitrogen rate. Agron. J. agj2.21101. doi: 10.1002/agj2.21101

[ref82] Walsh O. S. Torrion J. A. Liang X. Shafian S. Yang R. Belmont K. M.. (2020). Grain yield, quality, and spectral characteristics of wheat grown under varied nitrogen and irrigation. Agrosystems Geosci & Env 3:104. doi: 10.1002/agg2.20104

[ref79] Weightman R. M. Davis-Knight H. (2008). Triticale as a low input cereal for alcohol production. II. Potential to reduce greenhouse gas emissions relative to bioethanol from wheat. Asp. Appl. Biol. 165–172.

[ref80] Yang Y. Liu H. Tian X. Du W. (2022). Lodging resistance and feeding quality of triticale and cereal rye lines in an alpine pastoral area of P. R. China. Agronomy J. 114, 1284–1297. doi: 10.1002/agj2.21012

[ref81] Zhang X. Davidson E. A. Mauzerall D. L. Searchinger T. D. Dumas P. Shen Y. (2015). Managing nitrogen for sustainable development. Nature 528, 51–59. doi: 10.1038/nature15743, PMID: 26595273

