# Supplementary material for: Optimizing water and nitrogen productivity of wheat and triticale across diverse production environments to improve the sustainability of baked products
Source: Front Plant Sci. 2022 Sep 2;13:952303. doi: 10.3389/fpls.2022.952303 (PMC9491324; doi:10.3389/fpls.2022.952303)
Supplement: Supplementary file 1 [file Image_1.pdf]

Optimizing water and nitrogen productivity of wheat and triticale across diverse production environments to improve the sustainability of baked products

Santiago Tamagno[1], Cameron M. Pittelkow[1], George Fohner[2], Taylor S. Nelsen[1],

Joshua M. Hegarty[1], Claudia E. Carter[3], Teng Vang[3] and Mark E. Lundy[1,4]

[1] Department of Plant Sciences, University of California, Davis, CA 95616, USA
[2] California Grain Foundation, Woodland, CA 95776, USA
[3] California Wheat Commission, Woodland, CA 95776, USA
[4] Division of Agriculture and Natural Resources, University of California, Davis, CA 95618

Corresponding authors: stamagno@ucdavis.edu; melundy@ucdavis.edu

Supplementary material

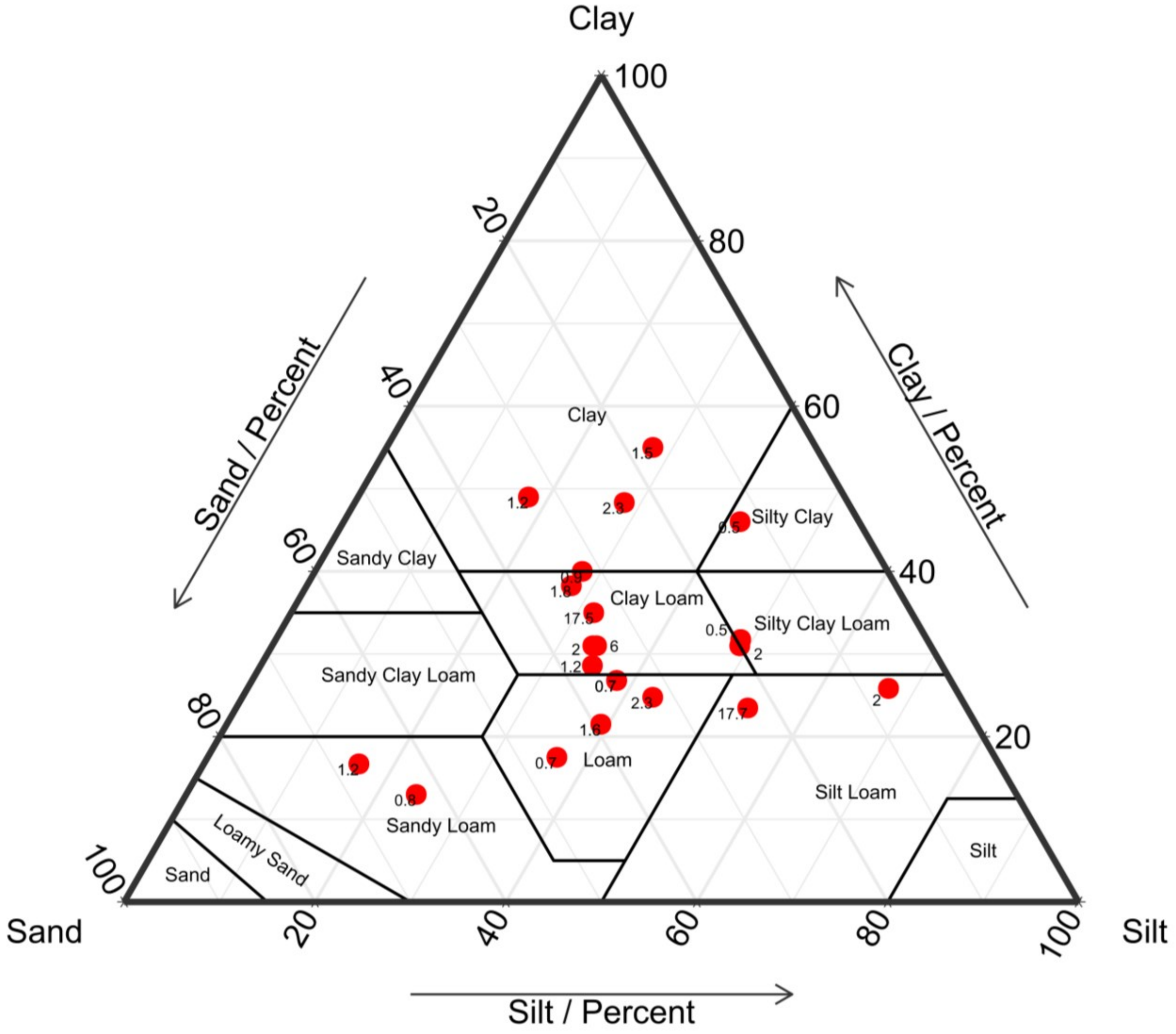

Supplementary Figure S1: Soil texture variability in the database, textural groups and organic matter. Each data point represents a site-year in the database. Numbers indicate the organic matter content (%).
